# 6-Benzyl-2-[(triphenyl-λ^5^-phosphanyl­idene)amino]-4,5,6,7-tetra­hydro­thieno[2,3-*c*]pyridine-3-carbonitrile

**DOI:** 10.1107/S1600536811035082

**Published:** 2011-09-03

**Authors:** Hong Chen, Kai Yan

**Affiliations:** a College of Chemistry and Life Science, China Three Gorges University, Yichang 443002, People’s Republic of China; b Hubei Key Laboratory of Natural Products Research and Development, China Three Gorges University, Yichang 443002, People’s Republic of China

## Abstract

In the title compound, C_33_H_28_N_3_PS, the P atom has a distorted tetra­hedral PNC_3_ environment, formed by the N atom and three aryl rings. No inter­molecular hydrogen-bonding inter­actions or π–π stacking inter­actions are present in the crystal structure.

## Related literature

For general background to the potential use of imino­phospho­ranes in the synthesis of *N*-heterocyclic compounds by means of an aza-Wittig reaction, see: Bräse *et al.* (2005 ▶); Ding *et al.* (2005 ▶); Huang *et al.* (2009*a* ▶,*b* ▶); Liu *et al.* (2008 ▶); Palacios *et al.* (2007 ▶). For a related structure, see: Muller (2011 ▶).
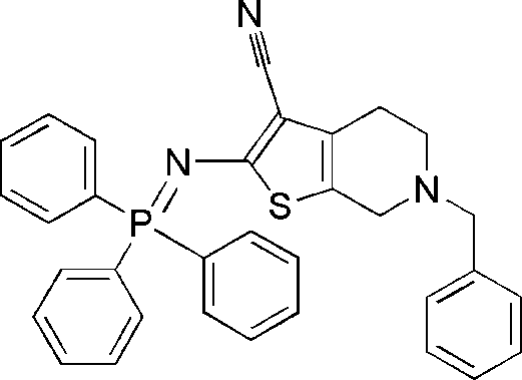


## Experimental

### 

#### Crystal data

C_33_H_28_N_3_PS
*M* *_r_* = 529.61
Monoclinic, 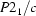

*a* = 8.926 (4) Å
*b* = 27.537 (12) Å
*c* = 11.719 (5) Å
β = 101.970 (4)°
*V* = 2818 (2) Å^3^
*Z* = 4
Mo *K*α radiation
μ = 0.20 mm^−1^
*T* = 296 K
0.23 × 0.15 × 0.14 mm

#### Data collection

Bruker SMART CCD diffractometer
Absorption correction: multi-scan (*SADABS*; Sheldrick, 1996 ▶) *T*_min_ = 0.965, *T*_max_ = 0.973
25582 measured reflections
6415 independent reflections
5506 reflections with *I* > 2σ(*I*)
*R*_int_ = 0.078

#### Refinement

*R*[*F* ^2^ > 2σ(*F* ^2^)] = 0.054
*wR*(*F* ^2^) = 0.145
*S* = 1.09
6415 reflections
343 parameters
14 restraints
H-atom parameters constrained
Δρ_max_ = 0.35 e Å^−3^
Δρ_min_ = −0.40 e Å^−3^

### 

Data collection: *SMART* (Bruker, 1997 ▶); cell refinement: *SAINT* (Bruker, 1997 ▶); data reduction: *SAINT*; program(s) used to solve structure: *SHELXTL* (Sheldrick, 2008 ▶); program(s) used to refine structure: *SHELXTL*; molecular graphics: *SHELXTL*; software used to prepare material for publication: *SHELXTL*.

## Supplementary Material

Crystal structure: contains datablock(s) I, global. DOI: 10.1107/S1600536811035082/aa2019sup1.cif

Structure factors: contains datablock(s) I. DOI: 10.1107/S1600536811035082/aa2019Isup2.hkl

Supplementary material file. DOI: 10.1107/S1600536811035082/aa2019Isup3.cml

Additional supplementary materials:  crystallographic information; 3D view; checkCIF report

## Figures and Tables

**Fig. 1.**
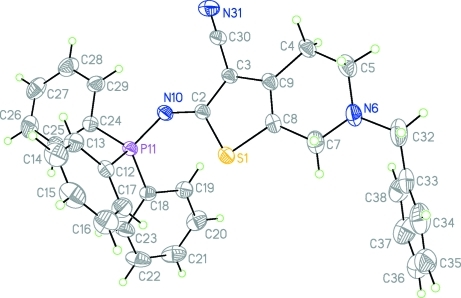
Molecular structure of the title compound with 50% probability displacement ellipsoids.

**Fig. 2.**
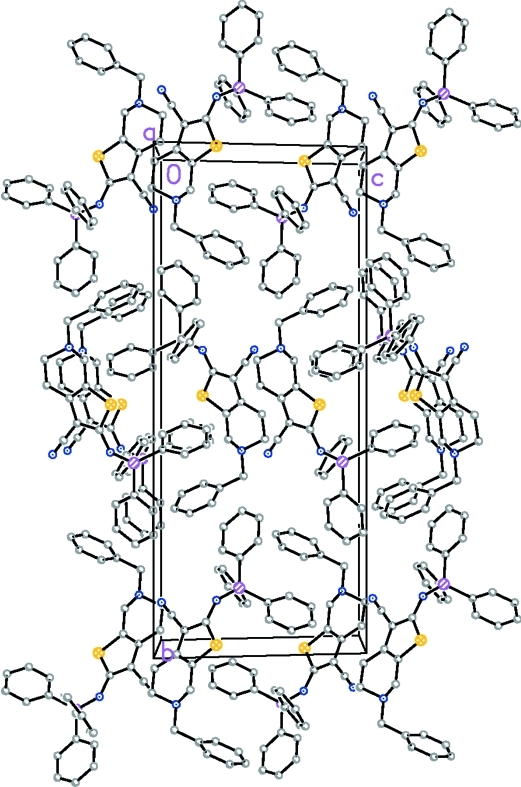
Packing diagram of the title compound projected along the c axis direction. H atoms are omitted for clarity.

**Fig. 3.**
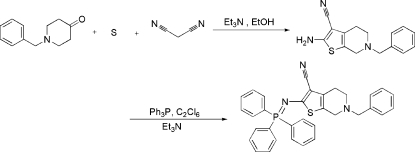
Reaction scheme.

## supplementary crystallographic information

### Comment

Over the past twenty years, the aza-Wittig reactions of iminophosphoranes have received increasing attention in view of their utility in the synthesis of N-heterocyclic compounds (Bräse *et al.*, 2005; Palacios *et al.*, 2007). Annelation of ring systems with N-heterocycles by means of an aza-Wittig reaction has been widely utilized because of the availability of functionalized iminophosphoranes. Consequently, the discovery of novel functionalized iminophosphoranes is important in this respect. Recently we have become interested in the synthesis of thienopyrimidinone, quinazolinones, and imidazolinones by an aza-Wittig reaction, with the aim of evaluating their fungicidal activities (Ding *et al.*, 2005; Huang *et al.*, 2009*a*,*b*; Liu *et al.*, 2008). Meanwhile, the title compound can be used as a new precursor for obtaining of bioactive molecules with fluorescence properties. Herein we wish to report the efficient synthesis, structural characterization of the title compound.

The molecular structure of the title compound is shown in Fig.1. The molecule has a benzyl substituent at the N6 atom of the thienopyridine ring and an nitrile group substituent at C3. Within the molecule, the bond lengths and bond angles present no unusual features. In the fused thienopyridine ring system, the thiophene ring is essentially coplanar, with maximum deviation of -0.0052 and 0.0059 Å for C8 and C9, respectively. The dihedral angle between plane (N6, C5, C7) and plane (C4, C5, C7) is 61.69°. The thiophene ring forms dihedral angles of 84.67, 73.58, 2.35 and 65.29° with the adjacent 6-membered rings C12–C17, C18–C23, C24–C29 and C33–C38, respectively. Meanwhile, the P atom has a distorted PNC_3_ tetrahedral environment, formed by the N atom [P═N = 1.5782 (16) Å] and three aryl rings. The crystal packing is determined by van der Waals forces. No intermolecular hydrogen bonding interaction or π-π stacking interactions are present in the crystal structure.

### Experimental

A well stirred mixture of 1-benzylpiperidin-4-one (1.89 g, 10 mmol), sulfur (0.32 g, 10 mmol), malononitrile (0.66 g, 10 mmol) in EtOH (10 ml) was cooled in an ice bath and treated dropwise with Et_3_N (1.01 g, 10 mmol). When addition was complete, the reaction mixture was warmed to 333 K for 40 min and then stored in the cold place until crystallization occurred. The product, 2-amino-6-benzyl-4,5,6,7-tetrahydrothieno[2,3-*c*]pyridine-3-carbonitrile (2.29 g, yield 85%) was recrystallized from EtOH as colourless needles, *M.p.* 422–423 K.

To a mixture of 2-amino-6-benzyl-4,5,6,7-tetrahydrothieno[2,3-*c*]pyridine-3-carbonitrile (1.35 g, 5 mmol), PPh_3_ (3.94 g, 15 mmol) and C_2_Cl_6_ (3.55 g, 15 mmol) in anhydrous CH_3_CN (40 ml), were added dropwise Et_3_N (2.42 g, 24 mmol) at room temperature. The color of the reaction mixture quickly turned yellow. After stirring for 4–6 h, the solvent was removed under reduced pressure and the residue was recrystallized from EtOH to give iminophosphorane in light yellow crystals, 3.63 g (83%), *M*.p. 463 K; IR (KBr), cm^-1^ 2190 (C≡N), 1490, 1346, 1100, 688; ^1^H NMR(CDCl_3_, 400 MHz) *δ*(*p.p.m.*): 7.78–7.23 (m, 20H, Ar—H), 3.62 (s, 2H, Ar—CH_2_), 3.24 (s, 2H, NCH_2_-thiophene), 2.75 (t, *J* = 8.7 Hz, 2H, NCH_2_CH_2_), 2.64 (t, *J* = 8.7 Hz, 2H, NCH_2_CH_2_); ESI-MS (*m/z*): 529.2 (*M*^+^), 530.2 ([*M*+H]^+^), 531.1 ([*M*+2H]^+^).

### Refinement

All H atoms were positioned geometrically [C—H = 0.93, 0.97 Å] and allowed to ride on their parent atoms, with *U*_iso_(H) = 1.2*U*_eq_(C). The rigid bond restraint "DELU, SIMU" instructions are used to restrain the anisotropic displacement parameters of C32—C33 and C34—C35 in the direction of the bond between them to be equal within a given standard uncertainty.

### Crystal data

**Table:**

C_33_H_28_N_3_PS	*F*(000) = 1112
*M**_r_* = 529.61	*D*_x_ = 1.248 Mg m^−^^3^
Monoclinic, *P*2_1_/*c*	Melting point: 463 K
Hall symbol: -P 2ybc	Mo *K*α radiation, λ = 0.71073 Å
*a* = 8.926 (4) Å	Cell parameters from 6648 reflections
*b* = 27.537 (12) Å	θ = 2.7–27.5°
*c* = 11.719 (5) Å	µ = 0.20 mm^−^^1^
β = 101.970 (4)°	*T* = 296 K
*V* = 2818 (2) Å^3^	Block, yellow
*Z* = 4	0.23 × 0.15 × 0.14 mm

### Data collection

**Table:**

Bruker SMART CCD diffractometer	6415 independent reflections
Radiation source: fine-focus sealed tube	5506 reflections with *I* > 2σ(*I*)
graphite	*R*_int_ = 0.078
CCD Profile fitting scans	θ_max_ = 27.5°, θ_min_ = 2.7°
Absorption correction: multi-scan (*SADABS*; Sheldrick, 1996)	*h* = −11→11
*T*_min_ = 0.965, *T*_max_ = 0.973	*k* = −35→35
25582 measured reflections	*l* = −15→15

### Refinement

**Table:**

Refinement on *F*^2^	Primary atom site location: structure-invariant direct methods
Least-squares matrix: full	Secondary atom site location: difference Fourier map
*R*[*F*^2^ > 2σ(*F*^2^)] = 0.054	Hydrogen site location: inferred from neighbouring sites
*wR*(*F*^2^) = 0.145	H-atom parameters constrained
*S* = 1.09	*w* = 1/[σ^2^(*F*_o_^2^) + (0.0593*P*)^2^ + 0.6222*P*] where *P* = (*F*_o_^2^ + 2*F*_c_^2^)/3
6415 reflections	(Δ/σ)_max_ = 0.001
343 parameters	Δρ_max_ = 0.35 e Å^−^^3^
14 restraints	Δρ_min_ = −0.40 e Å^−^^3^

### Special details

**Table:**

Geometry. All e.s.d.'s (except the e.s.d. in the dihedral angle between two l.s. planes) are estimated using the full covariance matrix. The cell e.s.d.'s are taken into account individually in the estimation of e.s.d.'s in distances, angles and torsion angles; correlations between e.s.d.'s in cell parameters are only used when they are defined by crystal symmetry. An approximate (isotropic) treatment of cell e.s.d.'s is used for estimating e.s.d.'s involving l.s. planes.
Refinement. Refinement of *F*^2^ against ALL reflections. The weighted *R*-factor *wR* and goodness of fit *S* are based on *F*^2^, conventional *R*-factors *R* are based on *F*, with *F* set to zero for negative *F*^2^. The threshold expression of *F*^2^ > σ(*F*^2^) is used only for calculating *R*-factors(gt) *etc*. and is not relevant to the choice of reflections for refinement. *R*-factors based on *F*^2^ are statistically about twice as large as those based on *F*, and *R*- factors based on ALL data will be even larger.

### Fractional atomic coordinates and isotropic or equivalent isotropic displacement parameters (Å^2^)

**Table:**

	*x*	*y*	*z*	*U*_iso_*/*U*_eq_	
S1	0.39018 (6)	0.490734 (17)	0.21538 (4)	0.04728 (14)	
P11	0.32811 (5)	0.375360 (16)	0.10480 (4)	0.03695 (13)	
C2	0.29442 (19)	0.44156 (6)	0.26335 (15)	0.0385 (4)	
N10	0.26996 (18)	0.39751 (5)	0.21202 (13)	0.0430 (3)	
C24	0.2666 (2)	0.31289 (7)	0.10029 (15)	0.0423 (4)	
C3	0.2455 (2)	0.45503 (7)	0.36345 (15)	0.0409 (4)	
C18	0.2416 (2)	0.40262 (7)	−0.03330 (16)	0.0433 (4)	
C9	0.2855 (2)	0.50376 (7)	0.40134 (15)	0.0431 (4)	
C4	0.2442 (3)	0.52902 (8)	0.50410 (17)	0.0533 (5)	
H4A	0.2822	0.5103	0.5742	0.064*	
H4B	0.1336	0.5310	0.4932	0.064*	
C12	0.5327 (2)	0.37745 (7)	0.11612 (16)	0.0431 (4)	
C8	0.3613 (2)	0.52716 (7)	0.32988 (17)	0.0467 (4)	
C30	0.1641 (2)	0.42171 (7)	0.42103 (16)	0.0482 (4)	
C19	0.1165 (2)	0.43269 (8)	−0.03768 (18)	0.0526 (5)	
H19	0.0819	0.4395	0.0302	0.063*	
N6	0.3003 (2)	0.60358 (7)	0.40430 (17)	0.0624 (5)	
C7	0.4035 (3)	0.57978 (8)	0.3403 (2)	0.0613 (6)	
H7A	0.5089	0.5835	0.3818	0.074*	
H7B	0.3932	0.5941	0.2635	0.074*	
C29	0.1868 (3)	0.29638 (8)	0.18196 (19)	0.0559 (5)	
H29	0.1703	0.3171	0.2409	0.067*	
N31	0.0979 (3)	0.39483 (8)	0.46615 (18)	0.0729 (6)	
C13	0.6239 (2)	0.34442 (9)	0.18892 (19)	0.0573 (5)	
H13	0.5789	0.3190	0.2221	0.069*	
C5	0.3120 (3)	0.58015 (9)	0.5189 (2)	0.0666 (6)	
H5A	0.2575	0.5994	0.5665	0.080*	
H5B	0.4187	0.5784	0.5586	0.080*	
C33	0.3007 (3)	0.67981 (8)	0.2956 (2)	0.0681 (6)	
C27	0.1534 (3)	0.21872 (9)	0.0894 (3)	0.0765 (8)	
H27	0.1140	0.1874	0.0849	0.092*	
C23	0.2916 (3)	0.39258 (10)	−0.13584 (18)	0.0643 (6)	
H23	0.3761	0.3726	−0.1341	0.077*	
C25	0.2919 (3)	0.28159 (8)	0.0134 (2)	0.0628 (6)	
H25	0.3471	0.2920	−0.0413	0.075*	
C26	0.2338 (4)	0.23443 (9)	0.0087 (2)	0.0775 (8)	
H26	0.2499	0.2134	−0.0498	0.093*	
C17	0.6018 (3)	0.41492 (9)	0.0675 (2)	0.0630 (6)	
H17	0.5420	0.4372	0.0184	0.076*	
C16	0.7602 (3)	0.41936 (11)	0.0916 (3)	0.0805 (8)	
H16	0.8063	0.4446	0.0587	0.097*	
C36	0.2460 (5)	0.72224 (11)	0.0748 (3)	0.0943 (9)	
H36	0.2282	0.7364	0.0012	0.113*	
C15	0.8486 (3)	0.38665 (12)	0.1639 (3)	0.0797 (8)	
H15	0.9546	0.3898	0.1803	0.096*	
C14	0.7818 (3)	0.34928 (11)	0.2122 (2)	0.0748 (7)	
H14	0.8427	0.3271	0.2607	0.090*	
C28	0.1311 (3)	0.24919 (9)	0.1766 (3)	0.0753 (7)	
H28	0.0786	0.2383	0.2324	0.090*	
C22	0.2153 (3)	0.41234 (12)	−0.2402 (2)	0.0827 (9)	
H22	0.2480	0.4053	−0.3087	0.099*	
C20	0.0417 (3)	0.45287 (10)	−0.1435 (2)	0.0723 (7)	
H20	−0.0416	0.4734	−0.1462	0.087*	
C21	0.0922 (3)	0.44216 (12)	−0.2433 (2)	0.0802 (8)	
H21	0.0420	0.4553	−0.3140	0.096*	
C35	0.3784 (4)	0.72998 (12)	0.1508 (4)	0.0936 (10)	
H35	0.4520	0.7499	0.1292	0.112*	
C32	0.3298 (4)	0.65581 (9)	0.4136 (3)	0.0824 (8)	
H32A	0.4353	0.6613	0.4526	0.099*	
H32B	0.2643	0.6704	0.4607	0.099*	
C38	0.1630 (3)	0.67270 (10)	0.2154 (3)	0.0808 (8)	
H38	0.0867	0.6536	0.2358	0.097*	
C34	0.4080 (3)	0.70931 (11)	0.2599 (3)	0.0888 (9)	
H34	0.5013	0.7152	0.3103	0.107*	
C37	0.1393 (4)	0.69361 (12)	0.1064 (3)	0.0931 (10)	
H37	0.0478	0.6879	0.0537	0.112*	

### Atomic displacement parameters (Å^2^)

**Table:**

	*U*^11^	*U*^22^	*U*^33^	*U*^12^	*U*^13^	*U*^23^
S1	0.0532 (3)	0.0445 (3)	0.0489 (3)	−0.00073 (19)	0.0216 (2)	−0.00419 (18)
P11	0.0375 (2)	0.0411 (2)	0.0327 (2)	0.00167 (17)	0.00837 (17)	−0.00069 (16)
C2	0.0377 (8)	0.0432 (9)	0.0346 (8)	0.0036 (7)	0.0076 (7)	0.0006 (6)
N10	0.0485 (8)	0.0442 (8)	0.0386 (8)	−0.0012 (6)	0.0143 (7)	−0.0042 (6)
C24	0.0422 (9)	0.0443 (9)	0.0374 (9)	0.0003 (7)	0.0014 (7)	−0.0019 (7)
C3	0.0432 (9)	0.0476 (9)	0.0314 (8)	0.0064 (7)	0.0065 (7)	0.0001 (7)
C18	0.0415 (9)	0.0501 (10)	0.0376 (9)	−0.0001 (7)	0.0070 (7)	0.0037 (7)
C9	0.0423 (9)	0.0505 (10)	0.0348 (9)	0.0091 (8)	0.0040 (7)	−0.0045 (7)
C4	0.0608 (12)	0.0609 (12)	0.0356 (9)	0.0127 (9)	0.0040 (9)	−0.0081 (8)
C12	0.0400 (9)	0.0491 (10)	0.0406 (9)	0.0035 (7)	0.0094 (7)	−0.0051 (7)
C8	0.0450 (9)	0.0462 (9)	0.0492 (10)	0.0052 (8)	0.0104 (8)	−0.0090 (8)
C30	0.0582 (11)	0.0536 (10)	0.0339 (9)	0.0080 (9)	0.0119 (8)	0.0017 (7)
C19	0.0443 (10)	0.0608 (12)	0.0512 (11)	0.0049 (9)	0.0064 (9)	0.0052 (9)
N6	0.0780 (13)	0.0471 (9)	0.0629 (11)	0.0041 (9)	0.0170 (10)	−0.0168 (8)
C7	0.0602 (12)	0.0494 (11)	0.0773 (15)	−0.0018 (10)	0.0213 (11)	−0.0146 (10)
C29	0.0691 (13)	0.0484 (11)	0.0515 (11)	−0.0074 (9)	0.0158 (10)	0.0022 (8)
N31	0.0974 (16)	0.0704 (13)	0.0583 (12)	−0.0061 (11)	0.0330 (12)	0.0098 (9)
C13	0.0485 (11)	0.0683 (13)	0.0529 (12)	0.0064 (10)	0.0056 (9)	0.0076 (10)
C5	0.0779 (15)	0.0678 (14)	0.0499 (12)	0.0049 (12)	0.0036 (11)	−0.0212 (10)
C33	0.0764 (15)	0.0455 (11)	0.0805 (16)	0.0092 (10)	0.0119 (13)	−0.0187 (10)
C27	0.0892 (18)	0.0464 (12)	0.0819 (18)	−0.0119 (12)	−0.0100 (15)	0.0005 (11)
C23	0.0621 (13)	0.0937 (17)	0.0391 (11)	0.0135 (12)	0.0152 (10)	0.0084 (10)
C25	0.0818 (15)	0.0564 (12)	0.0504 (12)	0.0022 (11)	0.0143 (11)	−0.0109 (9)
C26	0.103 (2)	0.0541 (13)	0.0662 (16)	0.0051 (13)	−0.0043 (14)	−0.0223 (11)
C17	0.0470 (11)	0.0652 (13)	0.0806 (16)	0.0025 (10)	0.0220 (11)	0.0123 (11)
C16	0.0498 (13)	0.0850 (18)	0.112 (2)	−0.0080 (12)	0.0288 (14)	0.0093 (16)
C36	0.119 (3)	0.0695 (18)	0.095 (2)	0.0008 (18)	0.025 (2)	−0.0063 (15)
C15	0.0403 (11)	0.114 (2)	0.0847 (19)	−0.0037 (13)	0.0122 (12)	−0.0092 (16)
C14	0.0482 (12)	0.105 (2)	0.0668 (16)	0.0173 (13)	0.0017 (11)	0.0068 (14)
C28	0.0913 (18)	0.0535 (13)	0.0814 (17)	−0.0172 (12)	0.0187 (14)	0.0079 (12)
C22	0.0768 (17)	0.132 (3)	0.0392 (12)	−0.0004 (17)	0.0118 (12)	0.0154 (13)
C20	0.0526 (12)	0.0819 (16)	0.0743 (17)	0.0134 (12)	−0.0057 (12)	0.0194 (13)
C21	0.0652 (15)	0.113 (2)	0.0549 (15)	−0.0024 (15)	−0.0054 (12)	0.0301 (14)
C35	0.105 (2)	0.0723 (18)	0.115 (3)	−0.0176 (17)	0.048 (2)	−0.0176 (17)
C32	0.1019 (19)	0.0533 (13)	0.0851 (17)	0.0030 (13)	0.0036 (15)	−0.0268 (11)
C38	0.0603 (14)	0.0636 (15)	0.116 (2)	−0.0038 (12)	0.0126 (15)	0.0062 (15)
C34	0.0697 (16)	0.0736 (17)	0.117 (3)	−0.0124 (14)	0.0048 (17)	−0.0369 (17)
C37	0.086 (2)	0.0790 (19)	0.101 (2)	0.0032 (16)	−0.0105 (18)	0.0071 (17)

### Geometric parameters (Å, °)

**Table:**

S1—C8	1.737 (2)	C33—C34	1.385 (4)
S1—C2	1.7550 (19)	C33—C38	1.397 (4)
P11—N10	1.5782 (16)	C33—C32	1.506 (4)
P11—C24	1.803 (2)	C27—C28	1.368 (4)
P11—C12	1.804 (2)	C27—C26	1.371 (4)
P11—C18	1.8057 (19)	C27—H27	0.9300
C2—N10	1.351 (2)	C23—C22	1.382 (3)
C2—C3	1.385 (2)	C23—H23	0.9300
C24—C29	1.383 (3)	C25—C26	1.395 (3)
C24—C25	1.388 (3)	C25—H25	0.9300
C3—C30	1.424 (3)	C26—H26	0.9300
C3—C9	1.435 (3)	C17—C16	1.389 (3)
C18—C19	1.383 (3)	C17—H17	0.9300
C18—C23	1.394 (3)	C16—C15	1.369 (4)
C9—C8	1.345 (3)	C16—H16	0.9300
C9—C4	1.501 (2)	C36—C35	1.341 (5)
C4—C5	1.528 (3)	C36—C37	1.347 (5)
C4—H4A	0.9700	C36—H36	0.9300
C4—H4B	0.9700	C15—C14	1.369 (4)
C12—C17	1.384 (3)	C15—H15	0.9300
C12—C13	1.389 (3)	C14—H14	0.9300
C8—C7	1.496 (3)	C28—H28	0.9300
C30—N31	1.143 (3)	C22—C21	1.367 (4)
C19—C20	1.396 (3)	C22—H22	0.9300
C19—H19	0.9300	C20—C21	1.370 (4)
N6—C7	1.458 (3)	C20—H20	0.9300
N6—C32	1.462 (3)	C21—H21	0.9300
N6—C5	1.474 (3)	C35—C34	1.373 (5)
C7—H7A	0.9700	C35—H35	0.9300
C7—H7B	0.9700	C32—H32A	0.9700
C29—C28	1.388 (3)	C32—H32B	0.9700
C29—H29	0.9300	C38—C37	1.376 (4)
C13—C14	1.386 (3)	C38—H38	0.9300
C13—H13	0.9300	C34—H34	0.9300
C5—H5A	0.9700	C37—H37	0.9300
C5—H5B	0.9700		
			
C8—S1—C2	92.17 (9)	C34—C33—C38	116.4 (3)
N10—P11—C24	104.09 (8)	C34—C33—C32	122.4 (3)
N10—P11—C12	115.09 (8)	C38—C33—C32	121.1 (3)
C24—P11—C12	109.28 (8)	C28—C27—C26	120.0 (2)
N10—P11—C18	113.76 (9)	C28—C27—H27	120.0
C24—P11—C18	107.42 (9)	C26—C27—H27	120.0
C12—P11—C18	106.90 (9)	C22—C23—C18	119.8 (2)
N10—C2—C3	124.56 (16)	C22—C23—H23	120.1
N10—C2—S1	126.48 (13)	C18—C23—H23	120.1
C3—C2—S1	108.96 (13)	C24—C25—C26	119.4 (2)
C2—N10—P11	130.67 (13)	C24—C25—H25	120.3
C29—C24—C25	119.31 (19)	C26—C25—H25	120.3
C29—C24—P11	119.39 (15)	C27—C26—C25	120.7 (2)
C25—C24—P11	121.24 (16)	C27—C26—H26	119.7
C2—C3—C30	120.69 (17)	C25—C26—H26	119.7
C2—C3—C9	114.23 (16)	C12—C17—C16	120.2 (2)
C30—C3—C9	125.07 (16)	C12—C17—H17	119.9
C19—C18—C23	119.18 (18)	C16—C17—H17	119.9
C19—C18—P11	118.30 (15)	C15—C16—C17	120.0 (3)
C23—C18—P11	122.44 (16)	C15—C16—H16	120.0
C8—C9—C3	112.36 (16)	C17—C16—H16	120.0
C8—C9—C4	121.09 (18)	C35—C36—C37	119.1 (3)
C3—C9—C4	126.47 (17)	C35—C36—H36	120.5
C9—C4—C5	111.13 (18)	C37—C36—H36	120.5
C9—C4—H4A	109.4	C16—C15—C14	120.4 (2)
C5—C4—H4A	109.4	C16—C15—H15	119.8
C9—C4—H4B	109.4	C14—C15—H15	119.8
C5—C4—H4B	109.4	C15—C14—C13	120.2 (2)
H4A—C4—H4B	108.0	C15—C14—H14	119.9
C17—C12—C13	119.13 (19)	C13—C14—H14	119.9
C17—C12—P11	121.62 (15)	C27—C28—C29	120.1 (3)
C13—C12—P11	118.64 (15)	C27—C28—H28	120.0
C9—C8—C7	124.33 (18)	C29—C28—H28	120.0
C9—C8—S1	112.26 (15)	C21—C22—C23	120.4 (2)
C7—C8—S1	123.18 (16)	C21—C22—H22	119.8
N31—C30—C3	179.3 (2)	C23—C22—H22	119.8
C18—C19—C20	120.3 (2)	C21—C20—C19	119.4 (2)
C18—C19—H19	119.8	C21—C20—H20	120.3
C20—C19—H19	119.8	C19—C20—H20	120.3
C7—N6—C32	110.9 (2)	C22—C21—C20	120.8 (2)
C7—N6—C5	109.80 (19)	C22—C21—H21	119.6
C32—N6—C5	112.64 (19)	C20—C21—H21	119.6
N6—C7—C8	107.58 (18)	C36—C35—C34	121.7 (3)
N6—C7—H7A	110.2	C36—C35—H35	119.1
C8—C7—H7A	110.2	C34—C35—H35	119.1
N6—C7—H7B	110.2	N6—C32—C33	111.7 (2)
C8—C7—H7B	110.2	N6—C32—H32A	109.3
H7A—C7—H7B	108.5	C33—C32—H32A	109.3
C24—C29—C28	120.5 (2)	N6—C32—H32B	109.3
C24—C29—H29	119.8	C33—C32—H32B	109.3
C28—C29—H29	119.8	H32A—C32—H32B	107.9
C14—C13—C12	120.0 (2)	C37—C38—C33	120.7 (3)
C14—C13—H13	120.0	C37—C38—H38	119.7
C12—C13—H13	120.0	C33—C38—H38	119.7
N6—C5—C4	110.37 (17)	C35—C34—C33	120.8 (3)
N6—C5—H5A	109.6	C35—C34—H34	119.6
C4—C5—H5A	109.6	C33—C34—H34	119.6
N6—C5—H5B	109.6	C36—C37—C38	121.2 (3)
C4—C5—H5B	109.6	C36—C37—H37	119.4
H5A—C5—H5B	108.1	C38—C37—H37	119.4
			
C8—S1—C2—N10	179.48 (17)	P11—C18—C19—C20	−177.18 (18)
C8—S1—C2—C3	−0.05 (14)	C32—N6—C7—C8	−177.4 (2)
C3—C2—N10—P11	−174.79 (14)	C5—N6—C7—C8	57.4 (2)
S1—C2—N10—P11	5.7 (3)	C9—C8—C7—N6	−22.4 (3)
C24—P11—N10—C2	173.39 (17)	S1—C8—C7—N6	151.68 (16)
C12—P11—N10—C2	53.8 (2)	C25—C24—C29—C28	−0.6 (3)
C18—P11—N10—C2	−70.01 (19)	P11—C24—C29—C28	176.58 (19)
N10—P11—C24—C29	0.25 (18)	C17—C12—C13—C14	0.1 (3)
C12—P11—C24—C29	123.69 (16)	P11—C12—C13—C14	−171.05 (19)
C18—P11—C24—C29	−120.68 (16)	C7—N6—C5—C4	−69.2 (2)
N10—P11—C24—C25	177.42 (17)	C32—N6—C5—C4	166.6 (2)
C12—P11—C24—C25	−59.14 (19)	C9—C4—C5—N6	39.9 (3)
C18—P11—C24—C25	56.49 (19)	C19—C18—C23—C22	−0.5 (4)
N10—C2—C3—C30	0.5 (3)	P11—C18—C23—C22	176.3 (2)
S1—C2—C3—C30	−179.95 (14)	C29—C24—C25—C26	1.3 (3)
N10—C2—C3—C9	179.89 (16)	P11—C24—C25—C26	−175.92 (18)
S1—C2—C3—C9	−0.57 (19)	C28—C27—C26—C25	−0.9 (4)
N10—P11—C18—C19	−11.62 (19)	C24—C25—C26—C27	−0.5 (4)
C24—P11—C18—C19	103.01 (17)	C13—C12—C17—C16	−0.2 (4)
C12—P11—C18—C19	−139.79 (16)	P11—C12—C17—C16	170.7 (2)
N10—P11—C18—C23	171.61 (18)	C12—C17—C16—C15	−0.1 (4)
C24—P11—C18—C23	−73.8 (2)	C17—C16—C15—C14	0.4 (5)
C12—P11—C18—C23	43.4 (2)	C16—C15—C14—C13	−0.5 (5)
C2—C3—C9—C8	1.1 (2)	C12—C13—C14—C15	0.2 (4)
C30—C3—C9—C8	−179.54 (17)	C26—C27—C28—C29	1.5 (4)
C2—C3—C9—C4	177.94 (17)	C24—C29—C28—C27	−0.8 (4)
C30—C3—C9—C4	−2.7 (3)	C18—C23—C22—C21	0.8 (4)
C8—C9—C4—C5	−5.3 (3)	C18—C19—C20—C21	0.8 (4)
C3—C9—C4—C5	178.09 (18)	C23—C22—C21—C20	−0.2 (5)
N10—P11—C12—C17	−94.91 (19)	C19—C20—C21—C22	−0.6 (4)
C24—P11—C12—C17	148.44 (18)	C37—C36—C35—C34	0.3 (5)
C18—P11—C12—C17	32.5 (2)	C7—N6—C32—C33	62.6 (3)
N10—P11—C12—C13	76.04 (18)	C5—N6—C32—C33	−173.9 (2)
C24—P11—C12—C13	−40.61 (18)	C34—C33—C32—N6	−127.8 (3)
C18—P11—C12—C13	−156.57 (16)	C38—C33—C32—N6	51.2 (3)
C3—C9—C8—C7	173.52 (19)	C34—C33—C38—C37	1.1 (4)
C4—C9—C8—C7	−3.5 (3)	C32—C33—C38—C37	−178.0 (3)
C3—C9—C8—S1	−1.1 (2)	C36—C35—C34—C33	−0.5 (5)
C4—C9—C8—S1	−178.14 (14)	C38—C33—C34—C35	−0.2 (4)
C2—S1—C8—C9	0.68 (15)	C32—C33—C34—C35	179.0 (3)
C2—S1—C8—C7	−174.02 (18)	C35—C36—C37—C38	0.7 (5)
C23—C18—C19—C20	−0.3 (3)	C33—C38—C37—C36	−1.4 (5)
